# RETIREMENT AND COGNITION: EXAMINING THE MENTAL RETIREMENT CONCEPT

**DOI:** 10.1093/geroni/igad104.3113

**Published:** 2023-12-21

**Authors:** Britney Veal, Hongdao Meng, Ross Andel

**Affiliations:** University of South Florida, Tampa, Florida, United States; University of South Florida, Tampa, Florida, United States; Arizona State University, Tempe, Arizona, United States

## Abstract

The concept of “mental retirement” posits that those approaching retirement experience less incentives to continue to engage with work, which is expressed as the so called “on-the-job” retirement effect, leading to negative changes in cognition. To test this notion, we set out to examine if participants who were about to voluntarily retire would have worse cognitive performance compared to those who continued working. We used members of the Health and Retirement Study who reported voluntarily retirement (n=333) in 2008 compared to those who continued working (n=2424). Cognition was assessed at four and two years prior to 2008 and in 2008. Analyses of covariance (ANCOVAs) were conducted with sociodemographic characteristics, chronic conditions, and baseline (2002) cognition as the covariates. Those who retired in 2008 and those who continued working varied on average age, 62.1±6.1 years vs. 60.7±6.1 years, p<.001, sex, 39% men vs. 45% men, p=.04, and average years of education, 13.3±2.6 years vs. 13.6±2.6, p=.04. ANCOVA results showed no significant between-group differences four years before retirement, F(1, 2669) = 0.00, p=.96, significantly worse cognitive scores among those who were planning to retire in two years compared to those who remained in the workforce, F(1, 2645) = 4.75, p=.03, and smaller, non-significant group differences in 2008, F(1, 2730) = 1.69, p=.19. These results support the existence of the “on-the-job” retirement effect, whereby the expectation of retired is accompanied by reduction in cognitive functioning, although it remains unclear whether this effect carries into retirement and/or under what circumstances.

